# Effect of Sodium Fluoride on the Properties of α-Hemihydrate Gypsum from Phosphogypsum

**DOI:** 10.3390/ma19091706

**Published:** 2026-04-23

**Authors:** Wanqing Zhou, Jiayi Huang, Rui Zou, Dongmei Liu, Jian Yang, Yi Qin, Yanzhou Peng

**Affiliations:** 1 Hubei Key Laboratory of Disaster Prevention and Mitigation, Yichang 443002, China; 2 College of Civil Engineering & Architecture, China Three Gorges University, Yichang 443002, China; 3 Hubei Engineering Research Center for Disaster Prevention and Mitigation (China Three Gorges University), Yichang 443002, China; 4 College of Civil Engineering & Architecture, Guangxi Eco-Engineering Vocational and Technical College, Liuzhou 545004, China

**Keywords:** α-hemihydrate gypsum, sodium fluoride, morphology, hydration, microstructure, setting time, strength

## Abstract

The presence of impurities directly affects the properties of α-hemihydrate gypsum (α-HH) prepared from phosphogypsum (PG) as a raw material. However, the effect of soluble fluorine impurities on the properties of α-HH by autoclaving remains insufficiently understood. This study investigated the influence of sodium fluoride on the morphology, hydration, and hardening properties of α-HH, using XRD, XPS, SEM, MIP, and tests of setting time, evolution of hydration temperature increase, and strength. The results showed that during the preparation of α-HH, some F^−^ reacted with Ca^2+^ to form CaF_2_, which adhered to the surface of the α-HH crystal, hindering the growth and development of the crystal and resulting in small crystals with rough surfaces. When α-HH hydrated, sodium fluoride caused the early, rapid nucleation of dihydrate gypsum (DH) crystals, accelerating the crystallization process of DH. The introduction of sodium fluoride inhibited the early hydration of α-HH and promoted its later hydration. The increase in sodium fluoride content caused the initial setting time of α-HH hydration to first increase and then decrease, while the final setting time continued to decrease. In the absence of sodium fluoride, the average pore diameter of the hardened paste was approximately 617.99 nm. When the NaF content was 0.2%, the DH crystals were prismatic and densely packed, which resulted in a decrease in the average pore diameter to 449.35 nm. When the NaF content was 0.6%, the DH crystals exhibited a plate-like morphology and were loosely interlocked, leading to an increase in the average pore diameter to 1169.58 nm. Based on these results, the sodium fluoride content in PG should be controlled below 0.2%.

## 1. Introduction

Phosphogypsum (PG) is a solid waste by-product generated from wet-process phosphoric acid production in the fertilizer industry [1,2], whose main component is CaSO_4_·2H_2_O (abbreviated as DH). PG also contains various harmful impurities, including phosphorus, phosphorus and fluorine compounds, insoluble phosphorus and fluorine compounds, and intercrystalline phosphorus compounds [3,4]. Because these impurities will affect the physical properties of gypsum and hinder the wide application of PG [3]. Therefore, various purification methods have been applied to remove the impurities from raw PG, such as washing, wet sieving, treatment with a mixture of silica and sulfuric acid or hot aqueous ammonium sulfate solutions, neutralization with lime, and thermal treatments. However, PG purification may increase the cost of the final product. With the rapid development of the phosphate fertilizer industry in China, China has become the world’s largest producer of wet-process phosphoric acid. The output of PG has been continuously increasing at a rate of about 70 million tons per year, and the current stockpile of PG has exceeded 900 million ton [5,6,7]. However, data from the China Phosphate and Compound Fertilizer Industry Association show that the current utilization rate of PG is only 40%. Large-scale PG stockpiling causes land occupation and considerable environmental pressure [8,9]. Thus, PG management and disposal have become major challenges for producers and authorities.

α-Hemihydrate gypsum (α-HH) is a high-strength gypsum with good working, environmental, and biological performance, widely used in precision casting, high-end building materials, arts and crafts, medical, and other fields [10]. Because the DH content in PG is very high, PG can be used as a raw material to produce α-HH by an autoclaved method, and the strength was mostly between 30 MPa and 40 MPa [11,12]. This utilization is an efficient way to realize high-value-added applications and sustainable development of PG, but the impurities, especially phosphorus, impair the properties of gypsum plaster and hinder the utilization of PG in the plaster industry [3,13,14,15,16]. To date, the effects and mechanism of soluble fluorine on the hydration, properties, and pore structure of β-hemihydrate gypsum (β-HH) have been well established. However, studies on the influence of soluble fluorine on the properties of α-HH remain limited. Song Jinbao [17] and Zhang Huan [18] separately prepared the DH samples containing different contents of soluble fluorine, then the samples were calcined to β-HH. The results indicated that during the calcination process, soluble fluorine has almost no effect on the dehydration process of DH. When β-HH hydrates, soluble fluorine causes a relatively low initial liquid phase supersaturation during the early stage of β-HH hydration and coarsens DH crystals; this leads to a shortened setting time and a decrease in strength. Note that α-HH is produced from DH using water or saturated steam, while β-HH is prepared by roasting dehydrated gypsum. There is a significant difference between α-HH and β-HH in the specific surface area, crystal size, imperfection, and surface topograph [19,20]. Therefore, the influence and mechanism of soluble fluorine on the composition, morphology, hydration, and strength of α-HH vary from that on β-HH. A recent study has shown that sodium fluoride alters the crystallinity, crystal habit, and crystal diameter of α-HH, causing some α-HH crystals to transform from fine needle-like shapes to aggregated sheet-like shapes [21]. It was inferred that sodium fluoride not only affects the crystal morphology and crystal shape regulation of α-HH but may also influence the hydration performance of α-HH. However, little attention has been paid to this.

In this work, α-HH was prepared with the autoclaving method, and the effect of sodium fluoride on the composition and morphology of α-HH was first investigated. Then, the experimental results of setting time, hydration temperatures, and anion concentration in the liquid phase of α-HH were tested. On these bases, the effect of sodium fluoride on the hydration properties of α-HH was discussed. In addition, the effect of sodium fluoride on the strength and microstructure of α-HH harden pastes was investigated. Based on the above results and analysis, the influencing mechanism of sodium fluoride on the properties of α-HH was revealed by simplifying the system, and the control range of sodium fluoride in PG used to produce α-HH was given. These results will provide the necessary theoretical and practical basis for the efficient application of PG in α-HH.

## 2. Materials and Methods

### 2.1. Materials

**Figure 1:**
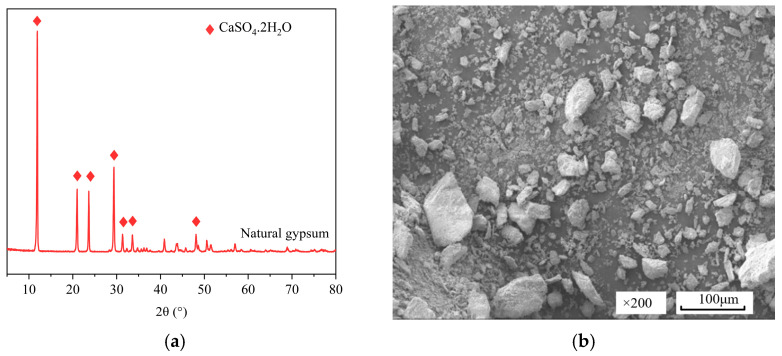
(**a**) XRD spectrum of NG; (**b**) SEM image of NG.

Natural Gypsum (NG), produced by Yingcheng Shengchang Gypsum Products Co., Ltd. (Yingcheng, China), appears as creamy yellow lumps. It was ball-milled with an SM-50 ball mill for 20 min, dried, and set aside. It can be seen from Figure 1a that the main chemical composition of NC is CaSO_4_·2H_2_O. Figure 1b shows a scanning electron microscope (SEM) image of the crystal morphology of NC, which appeared to be irregularly granular. The chemical reagents used include C_4_H_6_O_4_ (Succinic Acid, Shanghai McLean Biochemical Technology Co., Ltd., Shanghai, China), NaF (Soluble Fluoride from Shanghai McLean Biochemical Technology Co., Ltd.), and Ca(OH)_2_ (Calcium Hydroxide from Tianjin Tianli Chemical Reagent Co., Ltd., Tianjin, China), all of which were analytically pure.

### 2.2. Preparation of α-HH

The α-HH was prepared using autoclave technology. First, the crystallizing agent, succinic acid, was dissolved in purified water. The amount of crystallizer added was set at 0.06% of the total gypsum mass, based on the mass percentage of the external dopant. Then, the solution was poured into natural gypsum doped with impurities (sodium fluoride as an impurity doped according to the gypsum mass percentage of 0.2%, 0.4%, and 0.6%) and was mixed thoroughly using the net slurry mixer. The resulting mixture was then loaded into a vessel and subsequently placed into a YZF2A-type autoclave (manufactured by Wuxi Xidong Building Material Equipment Factory, Wuxi, China). Subsequently, the sample was autoclaved at 144 °C for 6 h to obtain a block of α-hemihydrate gypsum. After completing the autoclaving process, the α-HH block was quickly moved to the 101-3AB electric blast drying oven (manufactured by Tianjin Sedaris Experimental Analysis Instrument Manufacturing Factory) and dried at 110 °C until a constant weight was achieved. Subsequently, it was milled by a 2500C multifunctional pulverizer (manufactured by Dongguan Fangtai Electric Appliance Co., Dongguan, China) and passed through a 0.125 mm square hole sieve to produce α-HH powder.

### 2.3. Methods

#### 2.3.1. XRD Test

NG and *α*-HH were scanned using a Bruker AXS X-ray diffractometer (D8Advance) from Bruker, Bremen, Germany. The XRD patterns obtained were compared with standard PDF cards to analyze the physical phase composition of the products and to discern the crystal structure. Rietveld refinement of the X-ray diffraction patterns was performed. The mass fractions of the crystalline phases in the samples were calculated from the refined scale factors of each phase, combined with their respective crystal structure parameters.

#### 2.3.2. SEM Test

In this experiment, the crystal phase of NG, α-HH, and hardened pastes was observed using a Japan Electronics JSM-IT300 scanning electron microscope (JEOL Ltd., Tokyo, Japan).

#### 2.3.3. XPS Test

The surface chemical composition of α-HH crystals was analyzed using a Shimadzu AXIS Supra X-ray photoelectron spectrometer (XPS) (Shimadzu, Kyoto, Japan) with an optimal energy resolution of 0.43 eV.

#### 2.3.4. Hydration Temperature Rise Evolution Test

Hydration kinetics were monitored using an eight-channel isothermal calorimeter (TAM Air, Thermometric, Veddige, Sweden). The test material underwent pre-treatment at a constant temperature of 20 °C for 24 h. The test involved weighing 200 g of α-HH sample, weighing the water according to the standard consistency, and mixing the water with the sample in a container of equal volume. The mixture was stirred at a uniform speed for 30 s, then the sensor probes were immediately inserted. Data were collected at intervals of 10 s.

#### 2.3.5. Test of pH Value and Concentration of Fluoride Ions in the Supernatant of α-HH

The pH of the supernatant of α-HH paste in the absence or presence of sodium fluoride was measured using a portable pH meter from Mettler Toledo (Greifensee, Switzerland). The fluoride ion content during the hydration of the supernatant α-HH was determined by the specifications [22] with a water–cement ratio of 5:1.

#### 2.3.6. Physical and Mechanical Properties Testing of α-HH

The standard consistency and setting time of α-HH were determined according to the methods outlined in “α type high-strength gypsum” [23] and “Determination of physical properties of pure paste” [24]. For the setting time test, the paste was prepared at standard consistency and measured using a Vicat apparatus (Guangnian Zhixin Co., Ltd., Zhejiang, China). The crystalline water content was measured by gravimetric analysis following “Determination of water of crystalline content” [25]. Approximately 2 g of the sample, pre-conditioned at 20 °C ± 3 °C and 65% relative humidity for 24 h, was heated to 230 °C ± 5 °C until constant weight, and the loss on ignition was recorded as the crystalline water content.

For the determination of dry compressive strength, paste specimens were prepared at standard consistency and cast into 40 mm × 40 mm × 160 mm prismatic molds. After demolding, the specimens were cured at 20 °C ± 2 °C and 90% ± 5% relative humidity for 24 h, followed by oven-drying at 40 °C ± 1 °C to a constant weight. The compressive strength was then tested in accordance with “α type high-strength gypsum” [23] and “Determination of mechanical properties” [26], using a universal testing machine at a loading rate of 2.4 kN/s.

#### 2.3.7. Mercury Intrusion Porosimetry (MIP) for α-HH

The pore structure characteristics of α-HH hardened specimens with varying sodium fluoride (NaF) contents were characterized using mercury intrusion porosimetry. The testing was conducted with an AutoPore IV 9500 (Micromeritics Instrument Corporation, Norcross, GA, USA) fully automatic mercury intrusion porosimeter.

## 3. Results and Discussion

### 3.1. Effect of Sodium Fluoride on the Composition and Microstructure of α-HH

#### 3.1.1. The Crystalline Water Content

**Figure 2:**
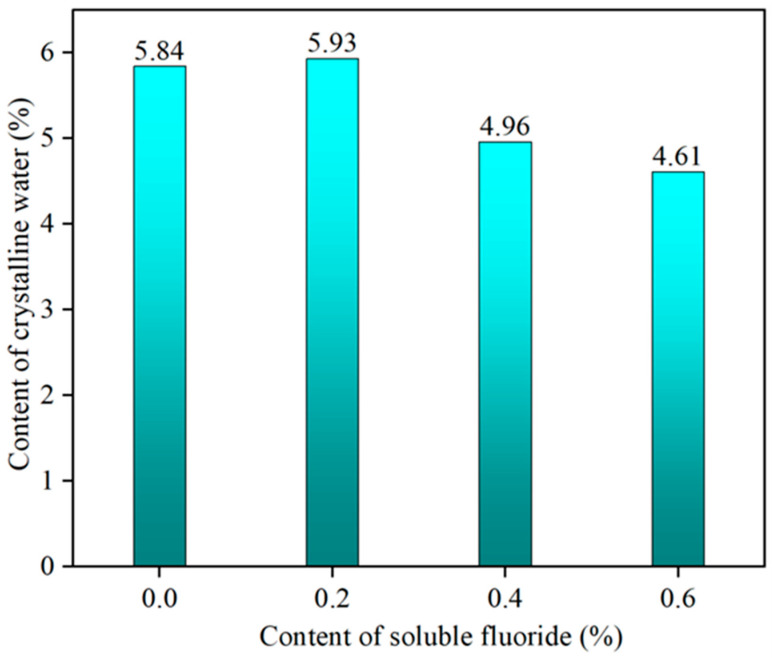
Crystalline water content of *α*-HH.

The effect of different levels of sodium fluoride on the crystalline water content of α-HH is shown in Figure 2. From Figure 2, it can be seen that the crystalline water content of α-HH increased first and then decreased with the increase in sodium fluoride, and the value of the crystalline water content was always lower than the theoretical value of 6.21% [27]. The results indicated that the α-HH included some III type anhydrite, which had been confirmed by the research of Li Ying [28]. With the increase in the sodium fluoride content, the content of α-HH decreased, while the content of III type anhydrite increased. The XRD pattern in Figure 4 also proved this conclusion.

#### 3.1.2. XRD

**Figure 3:**
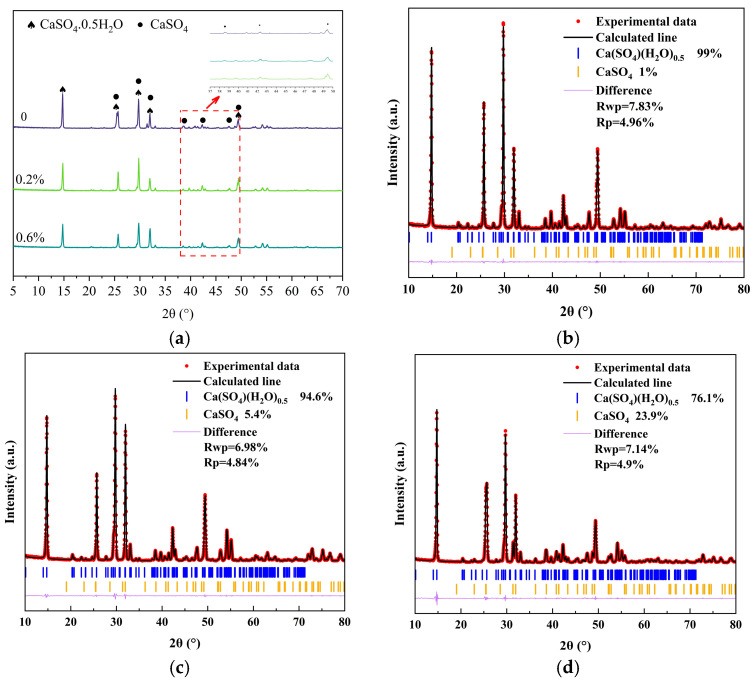
XRD patterns and Rietveld refinement of α-HH with different NaF contents: (**a**) XRD patterns of samples with different dosages; (**b**) Rietveld refinement of the blank group; (**c**) Rietveld refinement of 0.2% sodium fluoride; (**d**) Rietveld refinement of 0.6% sodium fluoride.

Figure 3 shows XRD patterns of α-HH in the presence of different levels of sodium fluoride. The characteristic peaks corresponding to α-HH and III type anhydrite can be observed in Figure 3. Compared with the control group, at sodium fluoride contentions of 0.2% and 0.6%, the intensity of characteristic peaks of α-HH and III type anhydrite was obviously enhanced. Quantitative phase analysis revealed that the control group consisted of 99% α-HH and 1% III type anhydrite. When the NaF concentration is 0.2%, the proportion of α-HH decreases to 94.6%, whereas the III type anhydrite increases to 5.4%. With the further addition of NaF to 0.6%, α-HH markedly drops to 76.1%, while III type anhydrite rises to 23.9%. The results indicated that as the sodium fluoride content rose, there was a decrease in the quantity of α-HH and an increase in the quantity of III type anhydrite. This conclusion was corroborated by the results of the crystalline water content analysis.

#### 3.1.3. SEM

**Figure 4:**
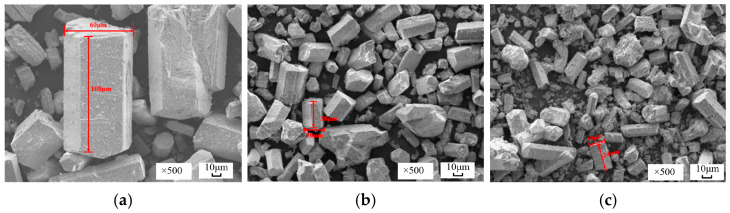
SEM images of *α*-HH in the presence of varying contents of sodium fluoride: (**a**) Blank group; (**b**) 0.2% sodium fluoride; (**c**) 0.6% sodium fluoride.

Figure 4 presents the SEM images of α-HH in the presence of varying concentrations of sodium fluoride. In the absence of sodium fluoride (the blank group), most of the well-crystallized -HH crystals manifested as short and stout hexagonal prisms, featuring a length-to-diameter ratio of 1.8. At 0.2% sodium fluoride, the well-crystallized crystals appeared as short and stout hexagonal prisms. Compared to the blank group, poorly grown and irregular crystals increased; the crystals became noticeably smaller and had an average length-to-diameter ratio of 1.9. At 0.6% sodium fluoride, the well-crystallized material exhibited a rod-like morphology and had an average length-to-diameter ratio of 2.4. There was a significant increase in poorly grown and irregular particles compared to the previous two groups, and the surface of the crystals became rough. The main reason was that the F^−^ in the sodium fluoride reacted with Ca^2+^ to form CaF_2_, which coated the surface of the α-HH crystals, hindering their growth, and resulting in smaller crystals with rough surfaces [16,29].

#### 3.1.4. XPS

**Figure 5:**
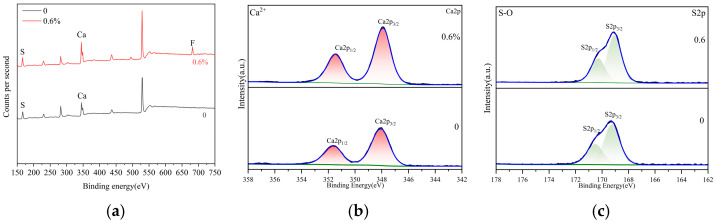
XPS spectra of *α*-HH at different sodium fluoride dosages: (**a**) Full spectra; (**b**) XPS spectra of Ca element; (**c**) XPS spectra of S element.

The XPS spectra of α-HH at various sodium fluoride dosages are shown in Figure 5. Figure 5a presents the full XPS spectra of α-HH in the absence and presence of sodium fluoride. For the blank group, the spectra exhibited signals for Ca, S, and O elements. When 0.6% sodium fluoride was added, the characteristic spectrum of the F element became visible. Figure 5b,c shows the XPS spectra of Ca and S elements in α-HH, both with and without sodium fluoride. At 0.6% sodium fluoride, the characteristic peaks of both Ca and S elements shifted to the left by 0.10 eV compared to the blank group. The shift in the Ca peak could be attributed to the reaction between F^−^ from the sodium fluoride and Ca^2+^ in natural gypsum, forming CaF_2_, which adsorbed onto the crystal surface of α-HH, as illustrated in Figure 4. Similarly, the S peak shifted to the left by 0.10 eV because Na^+^ from the sodium fluoride reacted with SO_4_^2−^ in natural gypsum to form Na_2_SO_4_ during the preparation of α-HH [19,30].

### 3.2. Effect of Sodium Fluoride on the Hydration Process of α-HH

#### 3.2.1. Hydration Temperature Rise Evolution

**Figure 6:**
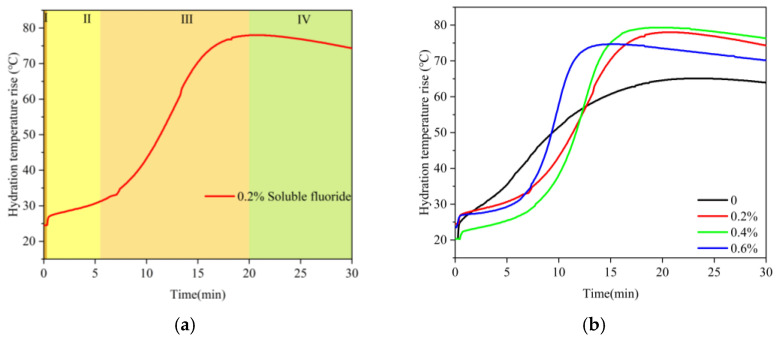
Effect of sodium fluoride on the hydration temperature rise of α-HH: (**a**) Stage division of the hydration temperature rise curve of α-HH; (**b**) hydration temperature rise curves of α-HH at different sodium fluoride dosages.

Figure 6 shows the effect of sodium fluoride on the hydration temperature rise of α-HH. As shown in Figure 6, the maximum temperature rise value (T_max_) during hydration in the blank group reached 65.1 °C, and the time for the maximum temperature rise value (t_max_) was 22 min. At 0.6% sodium fluoride, the T_max_ reached 74.7 °C, which was 12.8 °C higher than that of the blank group, and t_max_ was advanced to 15 min. It could be concluded that the T_max_ of α-HH was raised and t_max_ was shortened as the content of sodium fluoride increased.

The hydration temperature rise curves could be divided into the following four stages: the dissolution stage (stage I), the induction stage (stage II), the acceleration stage (stage III), and the stable stage (stage IV) [19,31,32]. Figure 6 shows that stage I of α-HH in the absence or presence of sodium fluoride was short, indicating that sodium fluoride had no obvious effect on stage I. There was no obvious stage II in the blank group, but stage III lasted 22 min. Compared to the blank group, stage II of α-HH in the presence of sodium fluoride was extended, and stage III was shortened. The author speculated that the reason for the extension of stage II was that, during the hydration of α-HH, CaF_2_ wrapped on the surface of the α-HH crystal, inhibited the dissolution and nucleation of DH, and led to a reduction in the number of crystals. In addition, during the preparation of α-HH, the sodium fluoride did not react completely to form CaF_2_, and the residual impurity remained in α-HH. The dissolution of α-HH was promoted, and the growth rate of DH crystals was increased in the presence of sodium fluoride, and the accelerating effect was more pronounced with the increase of soluble fluoride impurity content [3,33], which resulted in the stage III being shortened. The increase in III type anhydrite in α-HH with the increase in sodium fluoride was also another reason for the shortening of stage III.

#### 3.2.2. Setting Time

**Figure 7:**
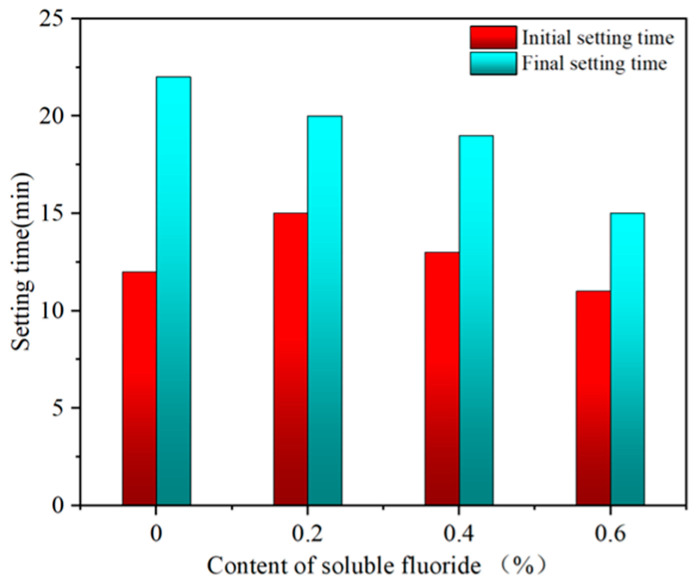
Effect of sodium fluoride on the setting time of *α*-HH.

The influence of sodium fluoride on the setting time of α-HH is shown in Figure 7. Figure 7 shows that when the sodium fluoride amount was 0, the initial and final setting times were 12 and 22 min, respectively. When the amount of sodium fluoride increased from 0.2% to 0.6%, the initial setting time increased first and then decreased, and the initial setting time of α-HH with 0.6% sodium fluoride was lower than that of the blank group, but the final setting time continued to decrease. This was because the presence of sodium fluoride delayed the nucleation of DH, but on the other hand, it promoted the growth rate of DH. When the delaying effect played an important role in the hydration of α-HH, the setting time was prolonged, but the setting time was shortened as the promotion effect acted mainly. Additionally, the increasing content of III type anhydrite in α-HH, resulting in a faster hydration rate, was another reason for the shortened final setting time. However, Guo [29] and Jia et al. [30] found that the initial and final setting times of hemihydrate gypsum decreased with the increase of sodium fluoride content compared with that of the blank group. This difference was due to the different incorporation methods of NaF as an impurity. Guo [29] and Jia et al. [30] incorporated NaF into the prepared hemihydrate gypsum, while in this paper, α-HH was prepared after NaF was incorporated into the raw material, which was more reliable.

### 3.3. pH and Fluoride Ion Concentration Analysis

**Figure 8:**
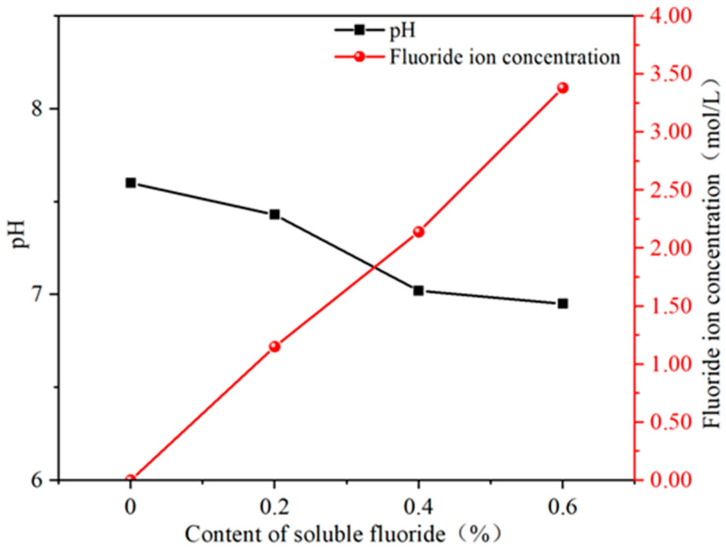
pH and fluoride ion concentration curves of the supernatant in the *α*-HH pastes.

The pH and fluoride ion concentration curves of the supernatant of α-HH paste in the absence or presence of sodium fluoride are depicted in Figure 8. Figure 8 shows that the pH of the blank group supernatant was 7.6. When the sodium fluoride content was 0.6%, the pH decreased to 6.8. The decline in pH was ascribed to the adsorption and desorption of OH^−^ and H^+^ on the interfaces of α-HH, DH, and CaF_2_ [34]. The concentration of fluoride ions in the supernatant gradually increased with an increase in sodium fluoride. The conclusion confirmed that some sodium fluoride remained in α-HH prepared from natural gypsum and dissolved during the hydration of α-HH. This conclusion was also consistent with the inference of the hydration temperature rise process.

### 3.4. Action Mechanism of Sodium Fluoride on the Properties of α-HH

When α-HH was prepared by autoclave technology using PG as raw material, the role of sodium fluoride in the properties of α-HH was described below. In a high-temperature and humid environment, 1.5 molecules of water of crystallization were shed from the lattice of DH to form the prismatic crystals of hemihydrate gypsum, which were dissolved in the liquid phase very quickly. When the concentration of hemihydrate gypsum in the liquid phase was saturated, hemihydrate gypsum crystallized rapidly [29]. Moreover, in this process, part of the sodium fluoride dissolved, and F^−^ combined with Ca^2+^ to generate CaF_2_, which adsorbed on the surface of α-HH crystals as shown in Figure 5. When the hydration reaction of α-HH occurred, α-HH quickly dissolved in water, and DH crystals nucleated and crystallized in the liquid phase. Sodium fluoride played a double role in the hydration process of α-HH. Firstly, CaF_2_ adsorbed on the surface of α-HH hindered its early dissolution. Secondly, the residual sodium fluoride in α-HH continually dissolved and reacted with Ca^2+^ to form CaF_2_, consequently fostering the dissolution of α-HH and the crystallization of DH. So, the initial setting time of α-HH increased first and then decreased, and the final setting time continuously decreased with the increase of the sodium fluoride. In addition, the α-HH in the presence of high sodium fluoride presented a layer that overlapped loosely and had low compressive strength.

### 3.5. Effect of Sodium Fluoride on the Strength of Harden Paste of α-HH

**Figure 9:**
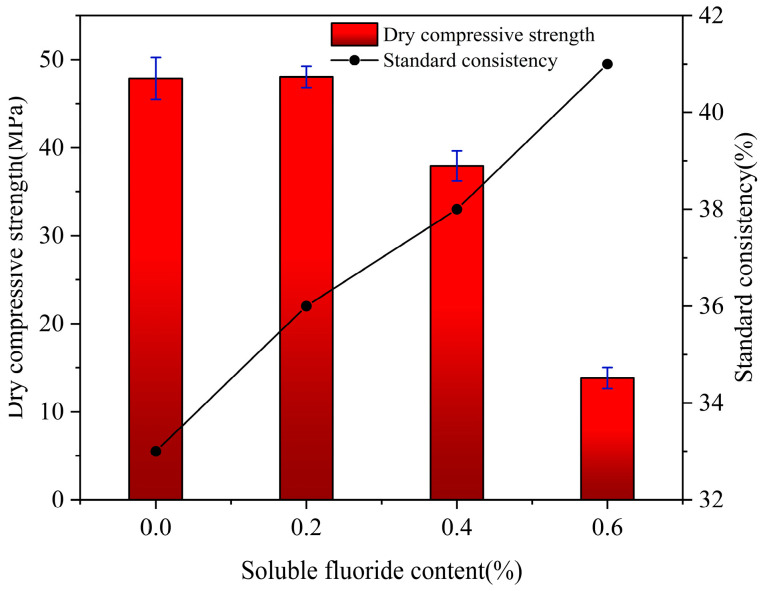
Effect of sodium fluoride on the standard consistency and the dry compressive strength of *α*-HH.

The standard consistency and dry compressive strength of α-HH in the presence of different levels of sodium fluoride are shown in Figure 9. From Figure 9, it can be seen that the standard consistency increased from 33% to 41% when the sodium fluoride increased from 0 to 0.6%. The crystal morphology of α-HH changed significantly, and the specific surface area increased, leading to an increase in the standard consistency. At the same time, the increasing content of III type anhydrite with high hydrophilicity also led to an increase in standard consistency. As can be seen from Figure 9, when the sodium fluoride was 0, the dry compressive strength of α-HH was 47.86 MPa, and when the sodium fluoride content impurity was 0.2%, the dry compressive strength of α-HH was 48.03 MPa, which had little change compared with the blank group. When the sodium fluoride content gradually increased to 0.4%, the dry compressive strength was 37.92 MPa. However, when the sodium fluoride content was 0.6%, the dry compressive strength decreased sharply to 13.83 MPa. Therefore, it could be concluded that when the content of sodium fluoride was equal to or greater than 0.4%, the dry compressive strength would be significantly decreased. Zhang Huan [18] and Santos et al. [34] observed that when hemihydrate gypsum was doped with sodium fluoride, the dry compressive strength of the hardened paste decreased, which was consistent with the research of this paper.

### 3.6. Effect of Sodium Fluoride on the Microstructure of Harden Paste of α-HH

**Figure 10:**
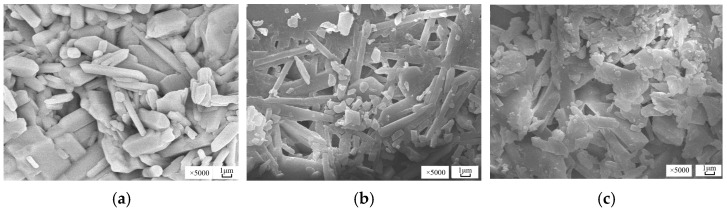
SEM pictures of hardened pastes with different levels of sodium fluoride: (**a**) Blank group; (**b**) 0.2% sodium fluoride; (**c**) 0.6% sodium fluoride.

The microstructure of hardened pastes is shown in Figure 10. Obviously, sodium fluoride had a significant effect on the morphology and size of DH grains. When the content of sodium fluoride was 0 or 0.2%, the DH grains were smooth, short, and prismatic. These grains were tightly stacked, and there were almost no holes in the hardened paste. At a soluble fluoride content of 0.6%, the DH grains appeared as plates and loosely overlapped with each other, resulting in increasing pores in the hardened paste. Zhang Huan [18] and Santos et al. [34] also observed that when α-HH was doped with soluble fluoride impurity, both lamellar crystals and rod-like dihydrate crystals were produced, and the generated lamellar crystals led to an increase in the pore size and the number of pores in the hardened pastes. During the hydration process of α-HH, sodium fluoride delayed the nucleation of DH, resulting in a decrease in the number of DH crystals, but meanwhile increasing the growth rate of DH crystals. As a result, the DH crystals were smaller and presented a layered structure [19].

**Figure 11:**
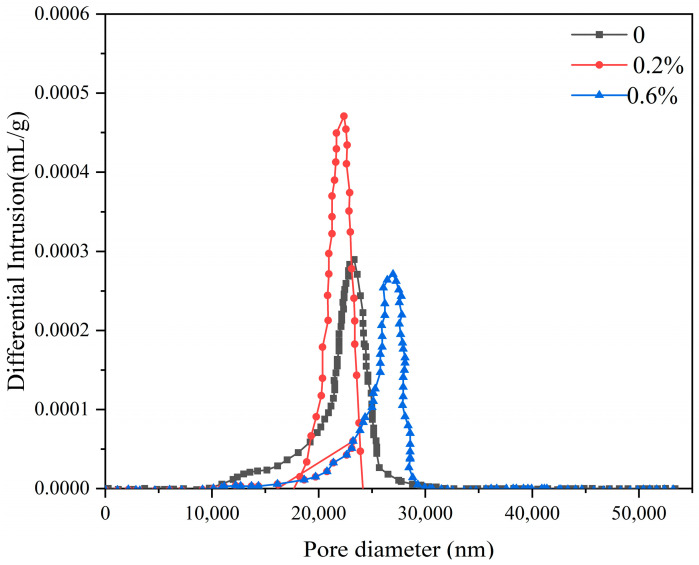
Pore size distribution curves of hardened pastes with different levels of sodium fluoride.

Figure 11 shows the pore size distribution curves of hardened α-HH pastes with varying NaF contents. As the NaF content increased from 0% to 0.6%, the average pore size of α-HH first decreased and then increased. In the absence of NaF, the average pore size was approximately 617.99 nm, with a relatively broad pore size distribution. At a NaF content of 0.2%, the average pore size decreased to 449.35 nm, and the pore size distribution became narrower. Upon further increasing the NaF content to 0.6%, the average pore size increased sharply to 1169.58 nm, and the distribution broadened considerably. The variation in pore size was primarily ascribed to changes in crystal morphology. When the NaF content was 0.2%, well-developed DH crystals were densely packed, resulting in a refined pore structure. When the NaF content was 0.6%, the DH crystals exhibited a plate-like shape and were loosely interlocked, resulting in a coarser and more porous structure. The pore structural deterioration and the increase in porosity were key factors contributing to the reduction in compressive strength.

## 4. Conclusions

In this study, the mechanism by which sodium fluoride influences the properties of α-HH was systematically investigated. Based on the experimental results and analyses, the main conclusions are as follows:

(1) With the increase in sodium fluoride content, the content of α-HH decreased, while the content of III type anhydrite increased. In the preparation process of α-HH, some F^−^ reacted with Ca^2+^ to form CaF_2_ adsorbed on the surface of α-HH crystals, which changed the morphology of α-HH crystals from short, stout, and smooth hexagonal prisms to rough, incomplete, and elongated rod-like shapes. In addition, the residual sodium fluoride remained in α-HH in the form of NaF.
(2) In the hydration process of α-HH, the dissolution of α-HH was hindered by CaF_2_ adsorbed on its surface, but the precipitation of DH was promoted obviously by the remaining sodium fluoride. The DH grains changed from a smooth and robust columnar shape to a flake crystal shape when the content of sodium fluoride increased from 0 to 0.6%.
(3) The initial setting time of α-HH increased first and then decreased; the final setting time continuously decreased with the increase in sodium fluoride.
(4) The dry compressive strength of α-HH with 0.2% sodium fluoride was similar to that of the blank group, but the strength in the case of sodium fluoride ≥ 0.6% decreased significantly.
(5) In the absence of sodium fluoride, the average pore diameter of the hardened paste was approximately 617.99 nm. When the NaF content was 0.2%, the DH crystals were prismatic and densely packed, which resulted in a decrease in the average pore diameter to 449.35 nm. When the NaF content was 0.6%, the DH crystals exhibited a plate-like morphology and were loosely interlocked, leading to an increase in the average pore diameter to 1169.58 nm. Therefore, considering both the mechanical properties and the microstructure, the sodium fluoride dosage should be controlled at 0.2% or below.

## Data Availability

The original contributions presented in the study are included in the article. Further inquiries can be directed to the corresponding author.
